# Cardiac Fibrosis Occurs before Arterial Hypertension Becomes Well Defined?

**DOI:** 10.5935/abc.20180243

**Published:** 2019-01

**Authors:** Claudio Pinho

**Affiliations:** Faculdade de Medicina da Pontifícia Universidade Católica (PUC) Campinas, Campinas, São Paulo - Brazil

**Keywords:** Myocites, Cardiac, Myoblasts, Cardiac, Prehypertension, Hypertension, Blood Pressue Monitoring Ambulatory/methods, Renin-Angiotensin System

Target organ damage (TOD) of systemic arterial hypertension (AH) in the heart modifies the cardiomyocyte, Interstice and its arteries. Alterations that occur in AH include cardiomyocyte hypertrophy, connective tissue hyperplasia and neovascularization stimulation, among others.^1^^-^^3^ These alterations depend essentially on the stimulus of the renin-angiotensin-aldosterone system (RAAS) and the sympathetic nervous system (SNS)^1^^-^^3^ which may not homogeneously impact Kidneys, heart, brain and blood vessels. The response of the connective tissue to AH induces collagen production by fibroblasts and consequently interstitial fibrosis.^1^^-^^3^

Since 2006 with the data published by Das MK et al.,^4^ we have started to correlate the presence of notches that form the fragmented QRS (fQRS) with non-homogeneous electrical conduction resulting from myocardial fibrosis which can be restorative or reactive.

With this information, Eyuboglu e Akdeniz^5^ proposed to correlate the presence of the fQRS and absense of the nocturnal decline in individuals with prehypertension, considering the existence of evidence of higher chances of TOD of AH in these cases.^3^^,^^5^^,^^6^

In the study population, Eyuboglu e Akdeniz^5^ , found 13.9% of fQRS, in spite of the small “n”. Moreover, these were statistically correlated to the absence of nocturnal decline of selected pre-hypertense through the ambulatory blood pressure monitoring (ABPM) without previous therapeutic approach, that is, without previous SRAA or SNS blockage. It was unclear in their methodology if the absence of the dipping referred to systolic blood pressure or diastolic blood pressure alone or both simultaneously. It was also unclear how those patients that didn’t sleep adequately due to pressure measures of ABPM were approached in the study. However, diurnal measurements showed levels compatible with prehypertension that may ease possible criticism.

Another item to be considered in Eyuboglu e Akdeniz^5^ is that although that study excluded left ventricular hypertrophy carriers identified by the echocardiogram and electrocardiogram, there would be interest in exploring the further correlation of mass index of VE /corporal surface area and fQRS since this would not be improbable.

Finally, the clinical relevance of the study is to alert us to the necessity of early treatment of the fQRS carriers, because they already show some reactive interstitial response, which is an evidence of early TOD in AH. We could also have in mind that the undertaking of the cardiomyocyte, interstice and vessels may not be simultaneous. Not even in terms of the response of AARS and SNS. Therefore in a group of pre-hypertense, the interstice could respond precociously with collagen production leading to fibrosis impacting not only diastolic function of left ventricle but also with repercussions on left atrium, overloading the contractile function before AH becomes evident. Therefore, cardiac fibrosis can occur before AH becomes evident!
